# Management of Acute Periodontal Abscess Mimicking Acute Apical Abscess in the Anterior Lingual Region: A Case Report

**DOI:** 10.7759/cureus.5592

**Published:** 2019-09-08

**Authors:** Omar A Alharbi, Muhammad Zubair Ahmad, Atif S Agwan, Durre Sadaf

**Affiliations:** 1 Conservative Dentistry, Qassim University, College of Dentistry, Buraydha, SAU

**Keywords:** periodontal abscess, antimicrobial agents, dental pulp test, dental pulp necrosis, apical suppurative periodontitis

## Abstract

Purulent infections of periodontal tissues are known as periodontal abscesses localized to the region of the involved tooth. Due to the high prevalence rate and aggressive symptoms, it is considered a dental emergency; urgent care is mandatory to maintain the overall health and well being of the patient. This case report describes the management of a patient who presented with an acute periodontal abscess secondary to poor oral hygiene. Clinically and radiographically, the lesion was mimicking an acute apical abscess secondary to pulpal necrosis. Periodontal treatment was started after completion of antibiotic therapy. The clinical presentation of the condition and results of the recovery, along with a brief review of relevant literature are discussed.

## Introduction

Periodontium, as a general term, describes the tissues surrounding and supporting the tooth structure. A localized purulent infection of the periodontal tissues adjacent to a periodontal pocket, also known as a periodontal abscess, is a frequently encountered periodontal condition that may be characterized by the rapid destruction of periodontal tissues [1-2]. The symptoms generally involve severe pain, swelling of the alveolar mucosa or gingiva, a reddish blue or red appearance of the affected tissues, and difficulty in chewing [1-3]. This condition may be acute or chronic, and its diagnosis is mainly based upon information from the patient history and clinical examination [3]. Pulp vitality tests, presence or absence of dental caries and deep periodontal pocket defects, radiographic examination, location of the abscess, and responsiveness to periodontal therapies are usually used to differentially diagnose a periodontal abscess from a lesion originating from the pulpal tissues of the affected tooth [4]. 

There are two main etiologies [2-6]. Firstly, in cases with a background of periodontitis, the condition is possibly due to untreated periodontitis or occurring during periodontal therapy [7]. Secondly, in cases that are not related to periodontitis, the main causes are usually the presence of foreign objects and radicular abnormalities [2, 8].

The prognosis of the involved tooth may be modified by the presence of an abscess. In a considerable number of cases, the condition is the reason for removal of the affected tooth. Hence, timely diagnosis with urgent management in such cases is recommended [7, 9-11].

Based on microbiological findings, mixed anerobic infections exist in periodontal abscesses. Candida species and herpes virus are sometimes found as well [4-6]. It is the third most common emergency dental infection accounting for 6%-7% of the prevalence across the world [2].

## Case presentation

In April 2018, a 58-year-old Saudi man (weight, 82 kg; height, 1.81m) with swelling, severe pain which was sharp in nature, refractory reddish gingiva, excessive bleeding from the gingiva, and tenderness to even slight tissue palpation on the mandibular anterior area presented to the dental clinics of Alrass Dental College, Qassim University, Buraydha, Saudi Arabia.

The patient was in good medical condition without the presence of any significant systemic disorders and had no history of food and drug allergies. He was a nonsmoker and nondrinker. He reported having a severe toothache for last two days in the mandibular anterior region and difficulty with normal eating, brushing, and speaking.

An extraoral examination revealed no facial abnormality. His temperature was 38.0°C. His blood pressure was 120/80 mmHg, and pulse was 75 beats/minute. Bilaterally, in the submandibular region, a few enlarged and tender lymph nodes were palpable.

An examination was performed intraorally that confirmed the presence of severe pain, tissue swelling, and bleeding from the gingiva.

**Figure 1:**
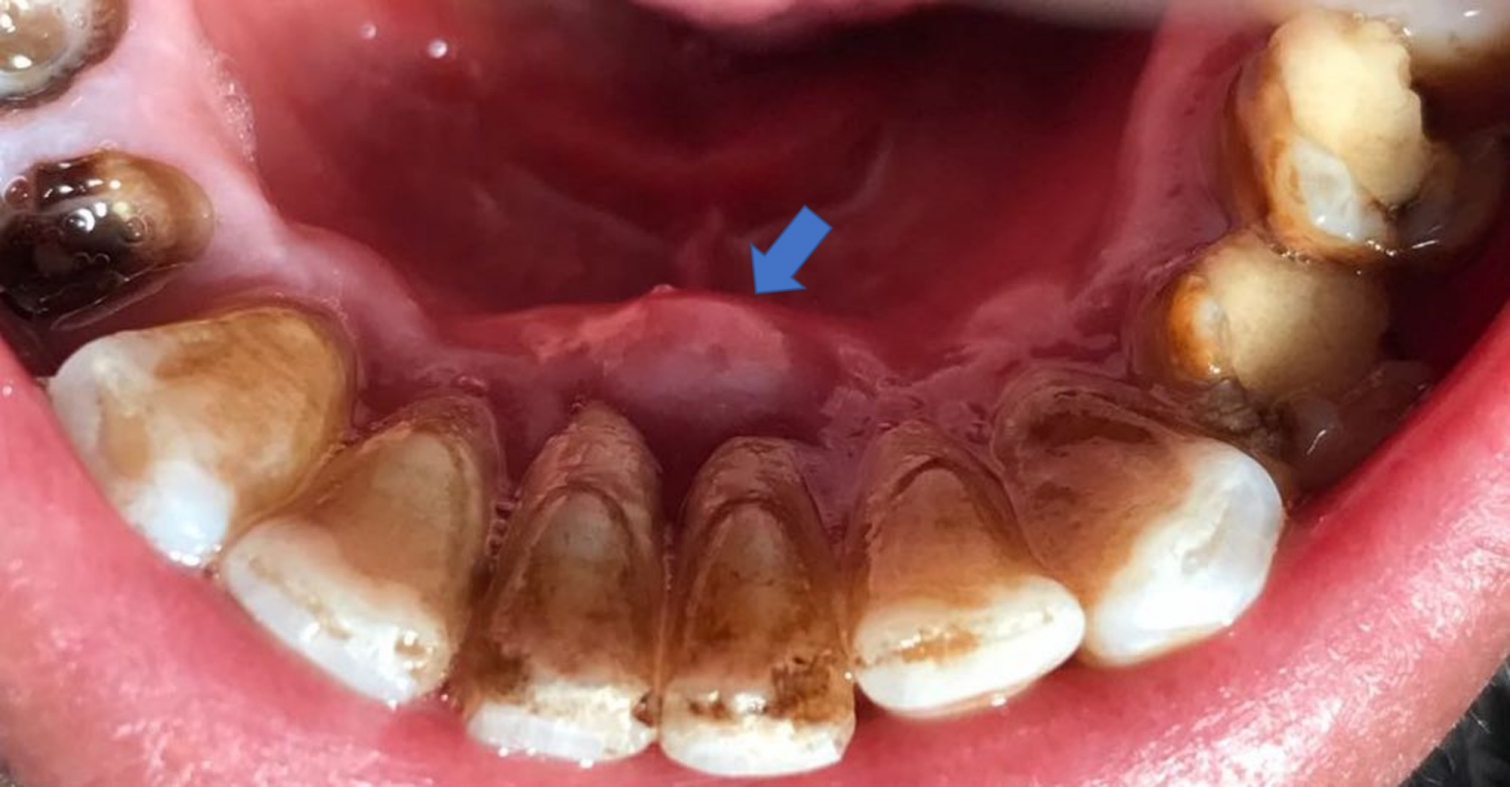
Clinical view of the periodontal abscess.

**Figure 2:**
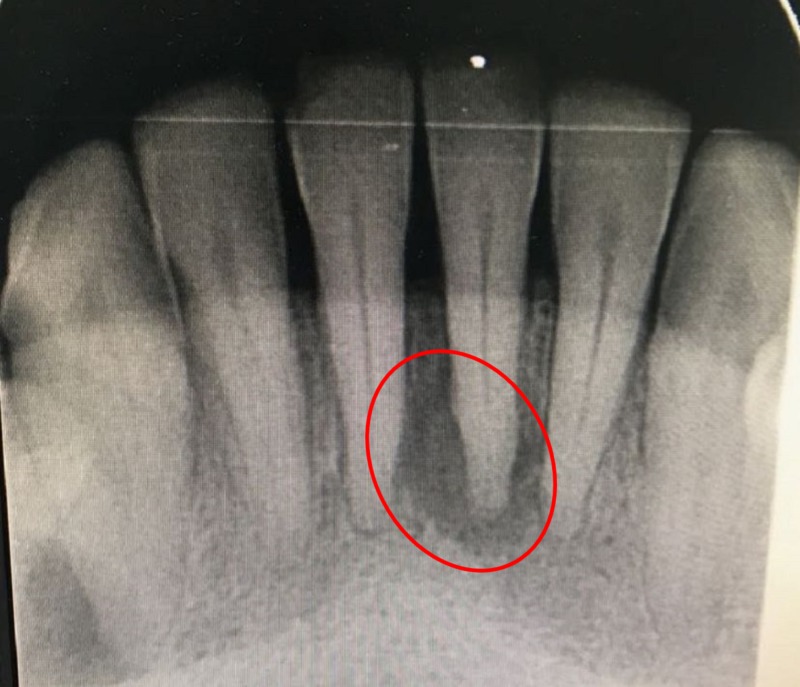
Periapical radiograph.

A heavy accumulation of dental plaque and calculus was noticed (Figure 1). Gingival recession on the lingual side was noted for the mandibular left central incisor. Periodontal pocket depths were normal (in the range of 3-4 mm) in rest of the dentition. A periapical radiograph revealed radiolucency around the apex of the mandibular left central incisor. Both central incisors had noncarious tooth surface loss on the disto-incisal surfaces of their respective crowns (Figure 2). Thermal and electric pulp sensibility tests were performed; all incisors in the mandible were found to be vital teeth.

After detailed clinical and radiographic examination, a diagnosis of an acute periodontal abscess in the mandibular left central incisor tooth was made.

After his periodontal examination was concluded, an irrigation was performed supra-gingivally using 0.2% chlorhexidine gluconate solution so that any local irritating factors, if present, could be removed as they may be the causative agents for his gingival inflammation. Antibiotics and analgesics were prescribed according to the guidelines as follows [12]: amoxicillin, 500 mg, thrice a day for seven days; metronidazole, 500 mg, thrice a day for seven days; naproxen, 550 mg, as needed.

The patient was prescribed a 0.12% chlorhexidine gluconate oral rinse twice daily for two weeks. The affected region was less painful after three days. At this point, scaling was carefully performed, and pus was drained from the abscess, then the patient was discharged.

**Figure 3:**
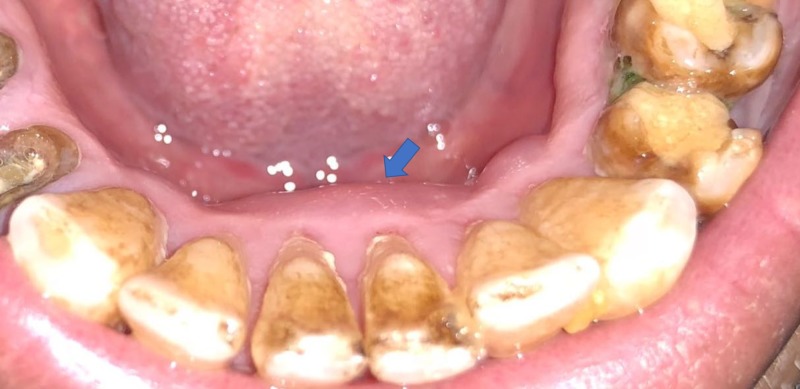
Postoperative results a week after therapy.

**Figure 4:**
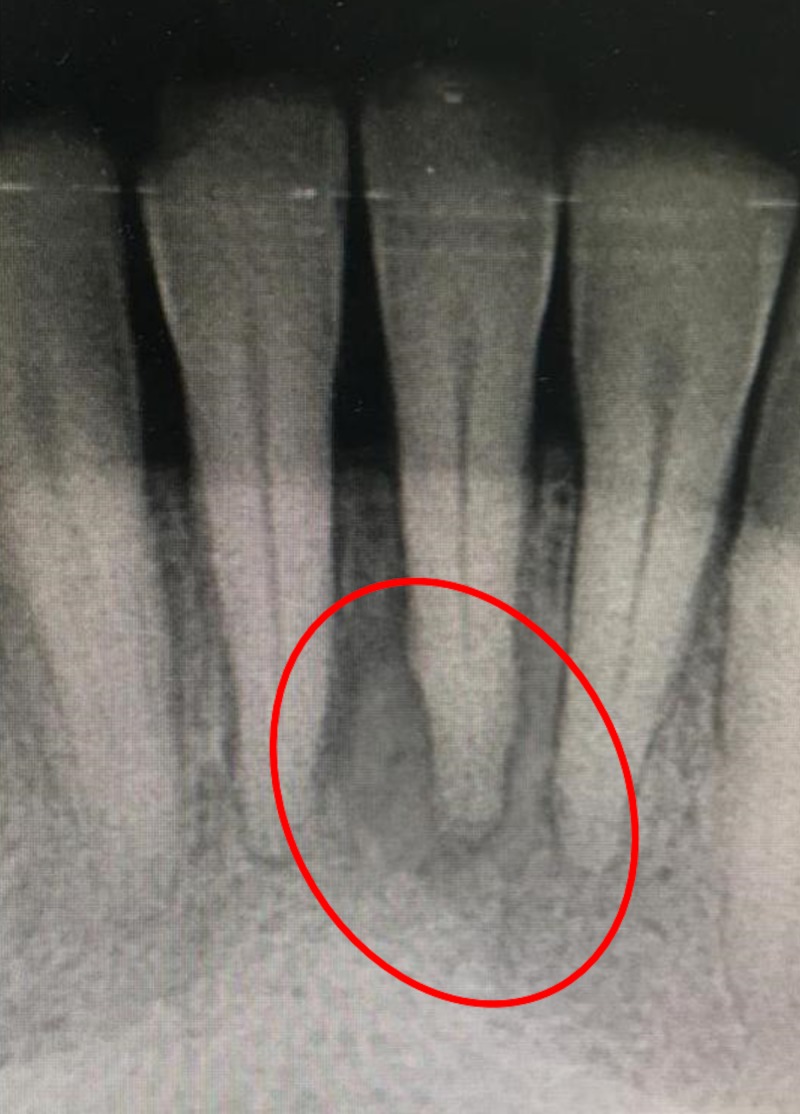
Periapical radiograph at three months follow-up.

One week following this procedure, complete healing of the affected area was noted; gingival reddishness was absent, there was no swelling, no lymphadenopathy. and no bleeding was found (Figure 3). A periapical radiograph at the three-month follow-up showed signs of healing in progress (Figure 4).

## Discussion

Periodontal abscesses have a very high prevalence rate of more than 20% of all the emergencies reported in dental clinics [13-14]. It is important to consider urgent care for this condition due to its predictable prognosis and to minimize the possibility of any further spread of infection [1-3, 15].

Substantial evidence exists favoring the use of antimicrobials systemically in cases of acute periodontal abscesses [16-18]. This is in contrast to acute apical abscesses of endodontic origin where extirpation of infected pulpal tissues with or without incision and drainage of associated inflamed soft mucosal tissues has proven to be the treatment of choice; in this case, there is no significant role for systemic antibiotics [19].

Regarding surgical management of acute periodontal abscesses, the literature is scant. Most of the information is provided in textbooks and case reports where empirical observations and expert opinions are the basis for conclusions [2-3, 9]. In this case, the periodontal abscess was related to excessive plaque deposits and extensive deposits of calculus. An acute periodontal abscess should be diagnosed after a detailed history, clinical and radiographic examination, and careful interpretation of all findings. Such conditions can be predictably treated with scaling, debridement, removal of local irritating factors such as calculus or bacterial plaque deposits, improving oral hygiene, giving systemic antibiotics, and standard surgical drainage [2, 9, 16, 19], as presented in the present case.

## Conclusions

It is highly advised that the diagnosis and subsequent treatment of acute periodontal abscesses should be done after an extremely careful interpretation of a detailed patient history and clinical and radiographic findings as the condition may mimic that of acute apical abscesses of endodontic origin. The decision on the use of systemic antimicrobials should be made solely on a case-to-case basis, and when a confirmed diagnosis cannot be made for reasons such as overshadowing of various other tooth- and patient-related factors, the use of systemic antibiotics should be avoided. It is important to provide the correct treatment of this pathological condition to maintain the health and integrity of periodontium.
